# Long-Term Health Expenditure Changes in Selected Balkan Countries

**DOI:** 10.3389/fpubh.2015.00152

**Published:** 2015-06-01

**Authors:** Nemanja Rancic, Aleksandra Kovacevic, Viktorija Dragojevic-Simic

**Affiliations:** ^1^Medical Faculty of Military Medical Academy, Centre for Clinical Pharmacology, University of Defence, Belgrade, Serbia

**Keywords:** health expenditure, Balkan, gross domestic product, life expectance, medicines

Spending on health is a significant policy issue for most countries’ economies of the world. Those expenditures put high pressure on public budgets, measuring government investments in health and well-being of their citizens. This task is even more challenging for most Balkan countries, embraced by the global economic crisis in the last decade and prior history of civil war for most of them.

Total health expenditure (THE), as a sum of public and private expenditure (1), expressed as percentage of gross domestic product (GDP), in the most of the selected Balkan countries, for the selected period from 1995 to 2012, showed obvious increase (Table 1) among majority of them. THE share of GDP among observed countries in 2012 was highest in Albania, with almost two and the half times larger share than in 1995. Romania, Serbia, and Bulgaria recorded about 1.5 fold higher share, while Greece, Bosnia, and Herzegovina and Montenegro remained at the same level as in 1995, comparing selected indicator. Exceptions were Croatia and The Former Yugoslav Republic of Macedonia (FYRM), with minor decrease. Possible explanations could be decline in the number of births, an increase in the mortality of younger aged people during the war and negative migration trends. Those could have influenced on stagnation of the growth of THE as percentage of GDP in Croatia (2), while FYRM is a country with more than 25% of the population living in poverty and with an unemployment rate in the country of over 30% (3). On the other hand, growth in health spending as a proportion of GDP contribute to the problem of economic and fiscal sustainability where health system revenue is insufficient to meet growing health obligations (4). This scenario is even more obvious in countries with shrinking economies.

**Table 1:** Expenditure data across selected Balkan countries – values reported to World Health Organization by the national authorities in 1995/2012*.

Countries/year	Total health expenditure (THE)% gross domestic product (GDP)		Total expenditure on health (current PPP int. $ per capita)		General government expenditure on health (current PPP int. $ per capita)		Private expenditure on health (current PPP int. $ per capita)	
	1995	2012	1995	2012	1995	2012	1995	2012
Albania	2.52	5.97	97.59	541.38	46.91	257.70	50.68	283.68
Bosnia and Herzegovina	9.37	9.89	128.43	927.61	47.81	659.99	80.62	267.62
Bulgaria	5.23	7.42	290.21	1177.08	214.64	662.58	75.57	514.50
Croatia	6.85	6.82	546.03	1409.77	472.08	1160.54	73.95	249.23
Greece	8.59	9.27	1263.09	2346.50	644.03	1584.04	574.40	762.46
Montenegro	7.42	7.57	445.18	1018.76	311.74	608.34	133.44	410.43
Romania	3.22	5.11	183.43	872.86	136.73	678.46	46.69	194.40
Serbia	7.32	10.47	259.86	1249.78	184.32	764.41	75.53	485.37
Slovenia	7.46	8.76	969.40	2419.86	753.22	1773.55	216.18	646.31
The Former Yugoslav Republic of Macedonia	8.51	7.13	421.39	834.94	247.17	535.15	174.22	299.79

*Data source: World Health Organization National Health Accounts Global Expenditure database http://apps.who.int/nha/database/Select/Indicators/en.

Total expenditures on health per capita, in terms of current purchase power parity in international $, in all selected Balkan countries showed significant growth comparing 1995 and 2012 (Table 1). Reason for this, not just local phenomenon, partially would be probably due to a population aging (5), which is in the close relationship with intensive health care spending and one of the cores of a health financing problem in Balkan countries (6). Life expectancy, as one of the widely used measure of health, showed increase in all observed countries, except Montenegro (7). The most obvious increase achieved Bosnia and Herzegovina, with prolonged 8 years of life in 2012 compared to 1995, reaching 76 years. Greece and Slovenia had the highest life expectancy, with 81 and 80 years, respectively (7).

In all observed countries, general governmental and private expenditures on health per capita also showed significant and constant growth (Table 1). However, general governmental expenditure still remains a dominant mode of the health financing in most selected Balkan countries. The only exception is Albania, where private expenditure on health in 2012 was slightly higher than governmental. The fact that the ratio of private expenditure growth in 2012 compared to 1995 was almost sevenfold higher in Bulgaria, closely followed by Serbia, Albania, and Romania remains. The greatest share of private expenditure consists of out-of-pocket expenditure, which accounts 56% of THE in Albania, 42% in Bosnia and Herzegovina, and 22% in Serbia and Montenegro (8). It could have severe effect on household economic status of already impoverished population. General governmental spending on health was highest in Slovenia, Greece, and Croatia, which are countries with highest total expenditure on health among all observed, as well. In Greece, private health insurance coverage remains low compared to other EU countries, despite dissatisfaction with the public system (9).

Expenditure on healthcare continues to grow, not only due to aging of populations but also due to growing public expectations on the accessibility and quality of healthcare (10). In well-developed economies, inpatient and professional services account for almost two-thirds of private health insurance spending, with pharmaceuticals share of 15% (11). Costs of increased use of technology, as well as of expensive medicines could escalate in the impending period (12). Structure of used medicines is also contributing to the healthcare costs. New brand-name drugs are often expensive, but the large number of generic drugs tends to increase the costs (13). Numerous factors may cause price increases for generic drugs: drugs shortages, supply disruptions, and consolidations within the generic drug industry (14).

We could conclude that, as countries become richer, the total amount of healthcare spending would increase. The model of health care financing mechanisms appears not to be a key driver for raising healthcare costs, as all observed countries showed similar growth in spite of relative different financing models among them.

## Conflict of Interest Statement

The authors declare that the research was conducted in the absence of any commercial or financial relationships that could be construed as a potential conflict of interest.
